# Vitamin C and *Helicobacter pylori* Infection: Current Knowledge and Future Prospects

**DOI:** 10.3389/fphys.2018.01103

**Published:** 2018-08-14

**Authors:** Haixin Mei, Hongbin Tu

**Affiliations:** ^1^Department of Gastroenterology, Xinyang Central Hospital, Xinyang, China; ^2^National Institute of Diabetes and Digestive and Kidney Diseases, NIH, Bethesda, MD, United States

**Keywords:** *Helicobacter pylori*, gastric diseases, vitamin C, concentration-function relationship, pharmacologic ascorbate, oral ingestion, I.V. administration, hydrogen peroxide (H_2_O_2_)

## Abstract

The gram-negative bacterium, *Helicobacter pylori* (*H. pylori*), infection is predominantly known for its strong association with development of gastric diseases, including gastritis, peptic ulcers, and stomach cancer. Numerous clinical reports show that ascorbic acid deficiency has been connect with gastritis. Vitamin C levels both in gastric acid and serum have constantly been affirmed to be low in subjects with *H. pylori* infected gastritis and peptic ulcers. Ascorbic acid supplementation likely relates to reduced incidences of bleeding from peptic ulcers and gastric cancer. *H. pylori* eradication is shown to increase vitamin C levels, while the benefits of ascorbic acid oral intake to increase the effectiveness of *H. pylori*-eradication therapy are controversial. Recent studies suggest that ascorbate intake intravenously, but not orally; pharmacologic ascorbate concentrations up to 30 mmol/L in blood, several millimolar in tissues as well as in interstitial fluid, are easily and safely achieved. Pharmacologic ascorbate can exert pro-oxidant effects locally as a drug by mediating hydrogen peroxide (H_2_O_2_) formation, which was applied to animal and clinical trials of cancer, sepsis, and severe burns etc. In this review, we summarize current understanding of the associations of vitamin C and *H. pylori* infection, and outline some potential strategies for *H. pylori* intervention from emerging advances on ascorbic acid physiology and pharmacology.

## Introduction

Since *Helicobacter pylori* (*H. pylori*) was first identified in 1982 by Robin Warren and Barry Marshall, gastritis and peptic ulcer disease have been gradually approached as an infectious disease (Warren and Marshall, 1983; Suerbaum and Michetti, 2002). As one of the most common bacterial infection factors, *H. pylori* infects more than 50% of the world's population (Taylor and Blaser, 1991). Most infected people remain asymptomatic; however, 10 ~ 20% *H. pylori* infection will ultimately develop into chronic gastritis, peptic ulceration, mucosa-associated lymphoid tumors, or even gastric adenocarcinoma (Warren and Marshall, 1983; Parsonnet et al., 1991; Wündisch et al., 2005). More important, eradication of *H. pylori* is an effective treatment for gastritis, peptic ulcer disease, and early lymphoma of mucosal-associated lymphoid tissue (MALT); it also has the potential to reduce the risk of gastric cancer development (Parsonnet et al., 1991; Ito et al., 2002; Wong et al., 2004; Wündisch et al., 2005).

Vitamin C is one of essential micronutrients for human health. Due to the accumulation of several mutations that turned gulonolactone oxidase into a non-functional pseudogene, humans, unlike most animals, have lost the ability to perform the crucial last step of vitamin C biosynthesis (Nishikimi and Yagi, 1991; De Tullio, 2010); instead we must obtain vitamin C from diet. Two major functions of vitamin C are as antioxidants and cofactors. As a co-factor, ascorbic acid donates electrons for at least 15 mammalian enzymes, including hydroxylase and monooxygenase involved in the synthesis of carnitine, collagen, and neurotransmitters (Levine et al., 2011; Padayatty and Levine, 2016). As an antioxidant, vitamin C protects the body from various deleterious effects of free radicals and reactive oxygen species (ROS) that are produced during normal metabolic processes, via active immune cells, as well as by exposure to toxins and contaminants (Carr and Frei, 1999). Low levels of vitamin C have been associated with many conditions, including scurvy, bleeding tendency, delayed wound healing, anemia, some cancers, infections, etc. (Naidu, 2003; Grosso et al., 2013; Padayatty and Levine, 2016). Regarding peptic ulcer disease and its complications, it is well known that ascorbic acid deficiency has been related to high occurrence of gastritis and bleeding from gastric and duodenal ulcers as well (Waring et al., 1996; Zhang et al., 1998; Aditi and Graham, 2012). Lower vitamin C levels, both in gastric juice and serum, have repeatedly been linked to patients with *H. pylori* infected gastritis and peptic ulcers (Ruiz et al., 1994; Zhang et al., 1998; Annibale et al., 2003). Normally, gastric gland ascorbate concentrations are three to seven times higher than plasma levels, indicating that ascorbic acid is actively transported or secreted from the plasma into the gastric juice (Annibale et al., 2003; Aditi and Graham, 2012). Ascorbic acid supplementations have been shown to be inversely related to gastric cancer (Zhang et al., 2002; Wong et al., 2004; Lam et al., 2013). *H. pylori* eradication can reverse the negative effect and increase vitamin C levels in serum and gastric juice; however, studies of ascorbic acid oral intake on *H. pylori*-eradication therapy reported ambiguous results (Sobala et al., 1993; Banerjee et al., 1994; Jarosz et al., 1998; Koçkar et al., 2001; Sezikli et al., 2012; Demirci et al., 2015).

We emphasize the importance of vitamin C concentration-function relationships in human health status. Vitamin C is playing different pathological, physiological, or pharmacological functions under the recognized reference range for plasma ascorbic acid concentrations of deficiency, healthy, or therapy dosage *in vivo* (Levine et al., 2011; Padayatty and Levine, 2016; Robitaille and Hoffer, 2016). Even with supplementation approaching maximally tolerated oral doses at 3–4 g, plasma ascorbate concentrations will just reach a plateau of about 200 ~ 300 μmol/L. In contrast, with intravenous ascorbate intake, pharmacologic ascorbate concentration of 25 ~ 30 mmol/L has been safely attained to treat various cancers, severe burns, sepsis, and other diseases (Tanaka et al., 2000; Nathens et al., 2002; Levine et al., 2011; Parrow et al., 2013; Wilson, 2013). The purpose of this review is to update the current knowledge of pharmacological vitamin C clinical data, associations of vitamin C and *H. pylori* infection, and the relevance it has in clinical use since ascorbic acid treatment on *H. pylori* eradication is yet to be fully understood.

## Current knowledge

### Vitamin C concentration-function relationship

A conception was proposed more than three decades ago that ideal vitamin intake is best determined based on biochemical, functional, and/or clinical outcome in relation to vitamin concentration (Levine, 1986). The concentration-function approach applying to vitamin C may be more desirable than recommended dietary allowance (RDA) for ascorbic acid, which was based on a safety margin to prevent deficiency (Levine et al., 2011). As shown in Table 1, there is an obvious causal chain of vitamin C concentration-function relationships among vitamin C intake, plasma ascorbate concentration, and relevant functional outcomes. Scurvy usually occurs when people consumed a diet with persistent lack of sufficient amounts of vitamin C (<10 mg daily), the diagnosis is confirmed by recording the plasma vitamin C concentration <11.4 μmol/L and observing the clinical improvement after appropriate oral vitamin C administration (Table 1; Lindblad et al., 2013; Robitaille and Hoffer, 2016). Low plasma ascorbate level, or hypovitaminosis C (plasma vitamin C concentration: 11.4 ~ 27 μmol/L) associated with a variety of disease complexes including cancer, sepsis, gastric ulcer, etc, may affect ~ 10% of the general population (Lindblad et al., 2013; Robitaille and Hoffer, 2016). Clinical data of *H. pylori* infected gastritis showed a typical example of vitamin C concentration-function relationship in Table 2, *H. pylori* infection was consistently associated with low vitamin C concentrations in the gastric juice before treatment, probably due to reduced intake, increased oxidation, and impaired or absent ascorbate secretion (Sobala et al., 1993; Banerjee et al., 1994; Ruiz et al., 1994; Farinati et al., 1996; Jarosz et al., 1998; Everett et al., 2001; Woodward et al., 2001; Henry et al., 2005; Tari et al., 2007). Vitamin C concentrations in gastric juice, but not in plasma, were improved significantly after *H. pylori* eradication (Table 2), it implied that *H. pylori* eradication recovers the normal transport or secretion of ascorbic acid from plasma into gastric juice. However, a large number of population-based surveys have shown that higher serum levels of ascorbic acid were associated with a decreased seroprevalence of *H. pylori* and especially of the pathogenic cagA-positive strain of *H. pylori* (Simon et al., 2003). In normal humans, vitamin C is vigorously transported into and concentrated in gastric juice; high concentration of ascorbate in gastric juice can inactivate and denature urease secreted by *H. pylori* at low pH mediated by H_2_O_2_ in the presence of Fe(3+) ions, preventing *H. pylori* survival and colonization into acidic stomach (Krajewska and Brindell, 2011; Pal et al., 2011).

**Table 1:** Vitamin C concentration-function relationship: Pathology - vitamin C deficiency or low level in diseases; Physiology - normal range of plasma vitamin C level: enzymatic cofactor and antioxidant; Pharmacology - high dose intravenous vitamin C administration: pro-oxidant effects.

**Plasma vitamin C concentration**	**Vitamin C intake**	**Physical condition**	**Function (biochemical or clinical outcomes)**
<11.4 μmol/L	~ <10 mg daily, P.O.	Pathological	Vitamin C deficiency, hypovitaminosis C, and diseases: scurvy, cancer, sepsis, gastric ulcer, and more diseases
27 ~ 100 μmol/L	<200 mg daily, P.O.	Physiological	Collagen, carnitine, and neurotransmitters synthesis; enzymatic cofactor- electron donor; antioxidant; support immune system etc.
mmol/L level	>1 g/injection, IV	Pharmacological	Pro-oxidant effects on disease treatment: cancer, bacterial or virus infections, burn, allergy, and more

*PO, per os; IV, intravenous*.

**Table 2:** Effect of *H. pylori* eradication on vitamin C concentration in gastric juice and plasma.

**Study**	**Intervention**	***H. pylori* eradication Subjects (n)**	**Vc assay**	**Vc conc. (μmol/L, B/A), plasma or gastric juice**	***P*-value**
Sobala et al., 1993	Antibiotic treatment	12	HPLC	44/45, plasma 34/57, gastric juice	NS 0.021
Banerjee et al., 1994	Antibiotic treatment	11	HPLC	15.3/15.9, plasma 13.6/63.6, gastric juice	NS 0.01
Ruiz et al., 1994	Antibiotic treatment	60	HPLC	44.3/43.2, plasma 32.4/48.8, gastric juice	NS 0.002
Farinati et al., 1996	Antibiotic treatment	10	HPLC	36.7/38.3, gastric juice	NS
Jarosz et al., 1998	Vc 5g 4 wks	8	Spectrophotometry	30.7/67.6, plasma 40.9/91.9, gastric juice	<0.01 <0.001
Everett et al., 2001	Antibiotic treatment	42	HPLC	29/38.6, plasma 38.04/72.1, gatric juice	0.3 <0.001
Tari et al., 2007	Antibiotic treatment	16	HPLC Spectrophotometry	23.7/38.1, plasma 17.3/77.8, gastric juice	0.0103 0.0021

*B/A, Before/after treatment; Vc, vitamin C; NS, non-significant*.

Normal dietary intake of ascorbic acid (≈ 40 mg per serving of fruits and vegetables, 2 ~ 5 servings daily) results in a recognized reference range for plasma ascorbic acid concentrations ranging from 27 to 100 μmol/L (Levine et al., 2001), which allows vitamin C to play its normal physiological role as enzymatic cofactors or antioxidant (Table 1). Ingestion of more vitamin C from foods will not produce higher concentrations *in vivo*, however, intravenous ascorbate administration produced plasma concentrations at millimolar level unachievable through oral administration (Padayatty et al., 2004; Chen et al., 2005; Parrow et al., 2013). Pharmacologic ascorbate can be used as a pro-drug for the formation of H_2_O_2_; the H_2_O_2_ concentration in extracellular fluid can reach as high as 200 μmol/L, which leads to production of large amounts of ROS inside or outside of cancer cells via iron mediated Fenton reactions and thus cause damage on macromolecules in cancer cells (Chen et al., 2007; Levine et al., 2011; Schoenfeld et al., 2017). The effects of pharmacologic ascorbate were further studied on clinical trials of cancer, bacterial or virus infections, burn, allergy, and so on (Tables 1, **4**, **5**).

**Figure 1:**
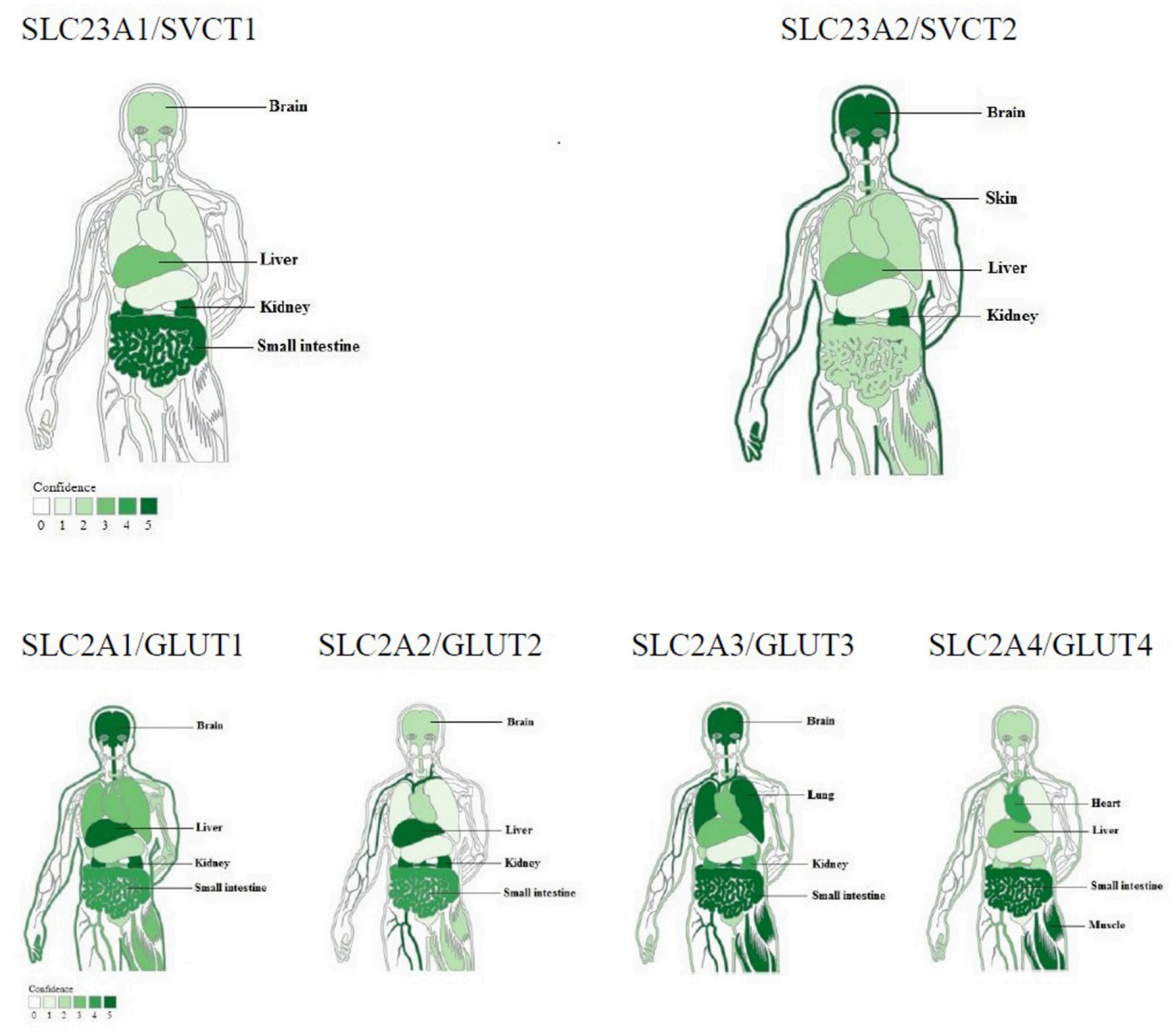
Tissue expression of ascorbic acid or DHA transporters responsible for tight control of vitamin C concentrations (modified from TISSUES: Tissue expression database). Ascorbic acid transporters: sodium-dependent vitamin C transporters 1 and 2. DHA transporters: Glucose transporter 1 ~ 4. Top four tissues with higher expression abundance were labeled for each transporter.

The clinical depletion-repletion pharmacokinetic data and other studies demonstrated that the concentrations of vitamin C in plasma and tissues were strictly controlled through intestinal absorption (bioavailability), tissue transport, renal reabsorption and excretion, and probably increased utilization in diseases (Levine et al., 2011). Cellular accumulation of vitamin C is due to transport of both ascorbic acid and its oxidized form (dehydroascorbic acid / DHA) *in vivo*. Ascorbate is transported via at least two sodium-dependent ascorbate transporters: SVCT1/Slc23a1 and SVCT2/Slc23a2 (Sotiriou et al., 2002; Corpe et al., 2010); SVCT1, which is confined to epithelial systems including liver, intestine, and kidney; and SVCT2, which is mainly expressed in brain, skin, kidney, lung, and placenta etc. (Figure 1, upper panel). Whereas DHA is primarily transported by facilitated glucose transporters, GLUT1 ~ 4, with different affinity and tissue expression abundance, and then reduced intracellularly to ascorbate immediately (Figure 1, low panel; Rumsey et al., 1997, 2000; Corpe et al., 2013). These transporter genes with elevated expression in particular tissues are closely related to their corresponding functions involved in the tight control mechanisms over vitamin C concentrations *in vivo* (Figure 1; Padayatty et al., 2004; Levine et al., 2011).

### Ascorbic acid supplement on *H. pylori* eradication: controversial data

Randomized trials have produced different results in the effects of vitamin C oral supplement on *H. pylori* eradication (Table 3). When supplemented vitamin C alone, only Jarosz et al. reported 29.6% *H. pylori* eradication rate in 5 g/day over 4 weeks vitamin C group, other reports showed that vitamin C oral intake had no effects on *H. pylori* eradication even with significant improvement of vitamin C concentration in plasma or gastric juice (Waring et al., 1996; Jarosz et al., 1998; Kamiji and Oliveira, 2005). When supplemented vitamin C along with standard antibiotic treatment on *H. pylori* infection, the results of *H. pylori* eradication were not consistent as well (Table 3). Some trials reported significant improvement on *H. pylori* eradication rate in antibiotic plus vitamin C groups compared to antibiotic groups (Chuang et al., 2007; Sezikli et al., 2009, 2011, 2012; Zojaji et al., 2009), but other studies showed no benefit from vitamin C addition (Koçkar et al., 2001; Chuang et al., 2002; Kaboli et al., 2009; Demirci et al., 2015).

**Table 3:** Vitamin C oral supplement on *H. pylori* infection (control group Vs Vitamin C+ group).

**Study**	**Regimen**	**Sample size (n)**	**Vc (mg/day) duration (wks)**	**Vc conc. (μmol/L) plasma or gastric juice**	**Eradication (%)**	***p*-value**
Waring et al., 1996	Unsupplement/Vc	11/12^N^ 32 ~ 36/19 ~ 20^G^	1000, 2	29/83, plasma 100/216, gastric juice 21/74, plasma 39/80, gastric juice	ND	<0.001^#^ <0.01^#^
Jarosz et al., 1998	Placebo/Vc	24/27	5000, 4	30.09/58.78, plasma 36.9/70.4, gastric juice	0/29.6	0.006^#^
Kamiji and Oliveira, 2005	Placebo/Vc	17/29	5000, 4	ND	0/0	NS
Koçkar et al., 2001	L,A,C/L,A,C+Vc	30/30	1000, 2	ND	66.7/50	NS
Chuang et al., 2002	L,A,M/L,A,M+Vc,Ve	49/55	500, 1 250, 6	ND	59.19/40	0.051
2007	O,A,C/O,A,C+Vc	55/61	1000, 1	ND	68/85	0.03
Kaboli et al., 2009	O,A,C500/ O,A,C250+Vc	100/114	500, 2	ND	89/86.8	0.623
Zojaji et al., 2009	O,A,M,B/O,A,M,B+Vc	162/150	500, 2	ND	48.8/78	<0.001
Sezikli et al., 2009	L,A,C+Vc,Ve/L,A,C	78/75	500, 4	ND	93.5/64	<0.005
2011	L,A,C+Vc,Ve/L,A,C	77/38	500, 4	ND	66.23/44.7	<0.005
2012	L,A,C+Vc,Ve/L,A,C	132/18	500, 4	ND	84/47.4	<0.05
Demirci et al., 2015	L,A,C/L,A,C+Vc,Ve L,A,C,B/L,A,C,B+Vc,Ve	84/84 89/91	500, 4	ND	75/71.4 80.9/83.5	0.728 0.792

N, No gastritis; G, Gastritis; L, lansoprazole; A, amoxicillin; C, clarithromycin; M, metronidazole; O, omeprazole; B, bismuth; Vc, vitamin C; Ve, vitamin E; ND, no data; NS, non-significant.

*p-value with #: Vitamin C concentration comparison in groups, others for eradication rate comparison*.

Although all these trials had high vitamin C dosage at 250 ~ 5,000 mg daily over 1 ~ 6 weeks with oral administration, plasma or gastric juice vitamin C concentrations were not reported in most of them (Table 3). Due to the above mentioned tight-controlled mechanisms, vitamin C daily doses for oral ingestion above 200 ~ 400 mg have no significant value for increasing vitamin C concentrations in plasma and tissues after reaching a plateau concentration of around 100 ~ 300 μmol/L (Levine et al., 1999, 2001). It thus made for a faulty experimental design for these trials without vitamin C concentration measurements (Table 3).

Several clinical attempts of intravenous injection with 500 or 1,000 mg vitamin C dosage were attempted to reverse initial low plasma or gastric juice ascorbic acid concentrations in ulcer patients. As early as in 1938, the effect of oral or intravenous administration of 1,000 mg of ascorbic acid on total body ascorbic acid stores were assessed; Portnoy and Wilkinson found that ulcer patients needed 3 ~ 4 times more amount of ascorbic acid intake to saturate body stores than normal controls (Portnoy and Wilkinson, 1938). In a case report, Sobala et al. IV injected a subject with 500 mg ascorbic acid at day 170 before *H. pylori* infection, day 37 and 161 after *H. pylori* infection respectively; it showed that the fasting gastric juice ascorbic acid rose rapidly at day170 sample, but was scarcely detectable at day 37 and remained low and rose only slightly at day 161 after the illness (Sobala et al., 1991). The effect of ethnicity, pH, and *H. pylori* infection on the changes of ascorbic acid concentration in gastric juice after intravenous injection of 500 mg vitamin C were examined; Correa et al. reported that 24 patients infected with *H. pylori* had a smaller but not statistically significant increase of ascorbic acid in gastric juice after intravenous injection (Correa et al., 1995).

Although the increased ascorbic acid in gastric juice was reported after IV injection, effects of ascorbic acid supplementation on *H. pylori* eradication were not mentioned in these studies (Portnoy and Wilkinson, 1938; Sobala et al., 1991; Correa et al., 1995). Notably, all these 500 or 1,000 mg vitamin C intravenous injections were administered only once, the dosage and duration of vitamin C IV injection may not be high and long enough there (Portnoy and Wilkinson, 1938; Sobala et al., 1991; Correa et al., 1995). Based on the conception of vitamin C concentration-function relationship, more clinical trials of pharmacological ascorbate on gastric ulcer and H. pylori eradication are warranted.

### Recent clinical use of pharmacological ascorbate on cancer and other diseases

The efficacy of vitamin C in cancer treatment has a controversial history over several decades. Observational-uncontrolled trials of pharmacologic ascorbate conducted by Cameron, Campbell, and Pauling on terminal cancer patients, given in mega doses of 10 g per day intravenously for 10 days and then orally administered 10 g/day indefinitely, suggested encouraging results including decreased tumor growth, increased survival time, and improved patient well-being (Cameron and Campbell, 1974; Cameron and Pauling, 1976, 1978). However, two well designed, double-blind placebo-controlled clinical trials undertaken at the Mayo Clinic on advanced cancer patients, treated with 10 g/day of ascorbate orally, showed no survival advantage at all (Creagan et al., 1979; Moertel et al., 1985). Unfortunately, these negative data led to the suspension of ascorbic acid as a potential cancer treatment, which was almost discarded by medical and scientific communities. Both teams treated patients with 10 g/day of ascorbic acid, the different routes of vitamin C administration, orally or intravenously, were the key and brought diametrically opposed in effects of cancer treatment. Based on the clinical depletion-repletion pharmacokinetic data, it is now clear that oral vitamin C produces a strictly controlled plasma concentration of μmol/L vitamin C; and the pharmacologic concentrations of vitamin C in the plasma at a level of mmol/L can only be achieved by parenteral administration, which bypassed such tight control mechanism (Levine et al., 1996, 2001, 2011; Padayatty et al., 2004; Parrow et al., 2013). Established by seminal studies by Chen et al. and with more *in vitro* and animal trial data from many laboratories (Padayatty et al., 2004; Chen et al., 2005, 2007, 2008; Yun et al., 2015; Schoenfeld et al., 2017), gradually, parenteral ascorbate for cancer treatment revitalized uneasily, with recent phase I or II clinical trials on various cancer types (Table 4).

**Table 4:** Recent clinical use of pharmacological Vitamin C on cancer treatments.

**Study**	**Regimen**	**Sample (n)**	**Vc dosage duration (months)**	**Plasma Vc conc. (mmol/L)**	**Clinical trial**	**Diseases**	**Effect**
Drisko et al., 2018	Vc	1	75–125 g 2–3 times per wk, ~ 48	Data not shown	Case report	PAD, stage IV	Body weight↑ tumor size↓liver lesions↓ survived ~ 4 yrs
Schoenfeld et al., 2017	Radiation therapy+ Temozolomide+Vc	11	15–87.5 g 3 times per wk, 9–11	≥20	phase I	GBM	safe and well tolerated PFS and OS↑
	CALGB + Vc	14	75 g twice per wk, ~ 3	16.4 ± 0.5	phase II	NSCLC, stage IIIB and IV	disease control rate, confirmed objective response rate ↑
Hoffer et al., 2015	Surgery Chemotherapy before, Vc	12	1.5 g/kg 2 or 3 times per wk, 1/3–19	10.8 ~ 19.6	phase I-II	#Advanced cancer	Nontoxic minor symptoms, 6 no objective anticancer response, 6 transient stable or longer-lastingstable diseases
Nielsen et al., 2015	Vc	10	5, 30, 60 g once a week, 1	1.8 ~ 19.3	phase II	Metastatic prostate cancer	Safe
Ma et al., 2014	Cp +Pax + Vc	10	15 g 1st shot, 75 or 100 g twice per wk, 12	20 ~ 23	pilot phase 1/2a	Ovarian cancer, stage III, IV	Disease progression/ relapse 8.75 months ↑ toxicities↓
Aldoss et al., 2014	ATO + Vc	11	1 g/day 5 days a wk, 1	Data not shown	Pilot study	non-APL AML	Limited antileukemia activity
Kawada et al., 2014	Vc after CHASER	3	15g 1st shot, 75 or 100 g every other day	>15	Phase I	NHL, stage III_B_, IV_A_	Safe, no obvious adverse effects
Stephenson et al., 2013	Vc	17	1 g/min 4 days/wk, 1	~ 49	Phase I	advanced cancer, stage I, III, IV	Minimal Adverse effects, no objective anticancer response
Welsh et al., 2013	Gemcitabine + Vc	9	15–125 g twice per wk, 2	≥20	Phase I	metastatic pancreatic cancer, stage IV	PFS and OS↑ Minimal Adverse effects
Monti et al., 2012	Gemcitabine + erlotinib + Vc	14	50–100 g 3 times per wk, 2	16.4, 27.8	Phase I	metastatic pancreatic cancer stage IV	tumor size ↓ Safe with adverse effects
Vollbracht et al., 2011	standard therapy+ Vc	53	7.5 g once a week, 4	Data not shown	Retrospective Cohort Study	BreastcancerUICC stages IIa to IIIb	side effects of disease and therapy↓ Safe
Hoffer et al., 2008	Chemotherapy before, Vc	24	0.4, 0.6, 0.9 1.5 g/kg 3 times per wk, 1	2.4, 4.7, 8.5, 11.3, 17, 26.2	Phase I	%advanced malignancy	Minimal Adverse effects and toxicity, no objective anticancer response
Padayatty et al., 2006	Nephrectomy before, Vc	1	65 g twice per wk, 10	Data not shown	Case report	RCC, nuclear grade III/IV	Complete remission
	Transurethral resection before, Vc	1	30 g twice per wk, 3; 30 g once every 1 ~ 2 months, 4 yrs			PBD, stage T2	Good health over 9 years
	Radiation therapy before, Vc	1	15 g twice per wk, 2; 15 g once to twice per wk, 7; 15 g once every 2–3 months, 1 yr			B-cell lymphoma stage III	Normal health over 10 years
Riordan et al., 2005	Chemotherapy before, Vc	24	150–710 mg /kg/day, 2	$Not accurate	Pilot study	Terminal cancer patients^*^	Progressive or stable Safe, minor side effects

PAD, Pancreatic ductal adenocarcinoma; GBM, glioblastoma; PFS, mean progression-free survival; OS, overall survival; CALGB, carboplatin + paclitaxel; NSCLC, Non-small-cell lung carcinoma; Cp, carboplatin; Pax, paclitaxel; ATO, Arsenic trioxide; non-APL AML, acute myeloid leukemia that excludes acute promyelocytic leukemia; CHASER, rituximab + cyclophosphamide + cytarabine + etoposide + dexamethasone; NHL, relapsed CD20-positive B-cell non-Hodgkin's lymphoma; RCC, renal cell carcinoma with lung metastasis; PBD, primary bladder tumor with multiple satellite tumors;#: most colon or rectal cancers, lung, and other cancer types; %: Urothelial, Head and neck, Sarcoma, Lymphoma, Prostate, Epidermoid, and other cancer types;

* *most colon or rectal primary tumors with metastasis; $: 2,6 dichlorophenolindophenol reduction method; Vc: Vitamin C; ↑ increase, ↓ decrease*.

As shown in Table 4, phase I or II trials, pilot studies, case reports, and retrospective cohort study of pharmacologic ascorbic acid on cancer treatment were published, including glioblastoma, B-cell lymphoma, non-Hodgkin's lymphoma, acute myeloid leukemia, breast cancer, non-small-cell lung carcinoma, metastatic pancreatic cancer, primary bladder tumor, renal cell carcinoma, metastatic prostate cancer, ovarian cancer, and other advanced malignancy (Riordan et al., 2005; Padayatty et al., 2006; Hoffer et al., 2008, 2015; Vollbracht et al., 2011; Monti et al., 2012; Stephenson et al., 2013; Welsh et al., 2013; Aldoss et al., 2014; Kawada et al., 2014; Ma et al., 2014; Nielsen et al., 2015; Schoenfeld et al., 2017; Drisko et al., 2018). All studies reported that intravenous vitamin C at dosage from 1g/day 5 days a week over 1 month to 75–125 g 2–3 times per week over 48 months, is generally safe, no toxicities, and well tolerated with minor adverse effects (Table 4; Aldoss et al., 2014; Drisko et al., 2018). Plasma vitamin C concentrations were measured and recorded from 1.8 to 49 mmol/L (Table 4; Stephenson et al., 2013; Nielsen et al., 2015), which are 30 ~ 600-fold higher than normal physical plasma ascorbic acid level. More important, when combined with standard cancer therapy and high dosage over a long period, intravenous ascorbic acid on some cancer types showed similar clinical benefits and improvement as before (Table 4). These positive results are prompting larger, longer-duration phase II or III clinical trials to determine susceptible cancer types, proper dosage, and precise clinical efficacy; such trials of pharmacologic ascorbate on advanced colorectal, gastric cancers are currently under way (NCT02969681; NCT03015675). To determine gastric cancer incidence and cause-specific mortality of 3,365 participants, in a masked factorial placebo-controlled trial with 14.7-year follow-up, Ma et al. reported that vitamins oral supplement (250 mg vitamin C, 100 IU vitamin E, and 37.5 μg selenium from yeast twice daily for a total of 7.3 years) had no significant effect on gastric cancer incidence and mortality (Ma et al., 2012). These negative results of oral vitamin C supplement on gastric cancer made the ongoing clinical trial of pharmacologic ascorbate on gastric cancer another good example to monitor (NCT03015675).

Pharmacologic ascorbate has also been widely used to treat and prevent many disorders like bacterial or virus infections, burns, allergies, and other diseases (Table 5). Intravenous vitamin C was given in doses of 1 or 2 g to 15 g per day, and plasma vitamin C concentrations could be reached 0.1 ~ 8.8 mmol/L (Table 5), which were one order of magnitude less than pharmacological ascorbate used in cancer treatments (Table 4). Clinical studies of patients with severe sepsis have found that intravenous vitamin C doses from 2.4 g over 1 h to 14 g/day over 4 days, increases total plasma nitrite, heart rate, cardiac index, and decreases the levels of pro-inflammatory biomarkers, SOFA scores, and mortality of ICU stay (Table 5; Galley et al., 1996; Fowler et al., 2014; Zabet et al., 2016; Marik et al., 2017). Two prospective trials of critically ill patients reported that standard therapy plus 3g/day intravenous ascorbic acid treatment reduced multiple organ failure, ICU stay length, and mortality rate as well (Table 5; Nathens et al., 2002; Sandesc et al., 2018). Two case reports of intravenous vitamin C injection, as adjuvant treatment for acute respiratory distress syndrome, showed reduced inflammation, increased alveolar fluid clearance, and even complete recovery (Table 5; Bharara et al., 2016; Fowler III et al., 2017). Pharmacologic ascorbate was also applied to treat virus infections like herpes zoster virus, hepatitis C virus, Epstein–Barr virus, and chikungunya virus, and most of them improved significantly (Table 5; Melhem et al., 2005; Schencking et al., 2010, 2012; Gonzalez et al., 2014; Mikirova and Hunninghake, 2014; Kim et al., 2016). Administrated 2 g ascorbic acid intravenously during myomectomy surgeries showed inconsistent effect of blood loss (Table 5; Pourmatroud et al., 2012; Lee et al., 2016). Given 66 mg/kg/h intravenous vitamin C to severe burn patients for the first day, they required less resuscitation fluid volume with more urine output (Table 5; Tanaka et al., 2000; Kahn et al., 2011). Furthermore, Hagel et al. found that intravenous infusion of 7.5 g of ascorbic acid could reduce the serum histamine concentrations in patients with infectious and allergic diseases (Table 5; Hagel et al., 2013).

**Table 5:** Intravenous Vitamin C used in infections, and other diseases.

**Diseases**	**Regimen**	**Sample (n)**	**Vc dosage duration (days)**	**plasma Vc conc. (μmol/L)**	**Effect**	**References**
Sepsis	Placebo/NAC + Ve + Vc	14/16	20 mg/kg/h plus bolus doses of 1 g, 1 h	<130	well tolerated Total plasma nitrite, heart rate, cardiac index↑ Systemic vascular resistance index↓	Galley et al., 1996
	Placebo/Lo–Vc/Hi-Vc	8/8/8	50 or 200 mg/kg/24 h, 4	18/300/3000	safe and well tolerated SOFA scores, inflammation, and endothelial injury↓	Fowler et al., 2014
	Placebo/Vc	14/14	25 mg/kg/6 h, 3	Data not shown	Norepinephrine dosage and duration↓ ICU stay mortality↓	Zabet et al., 2016
	Standard/standard + hydrocortisone + thiamine + Vc	47/47	1.5 g every 6 h, 4 days or until ICU discharge	Data not shown	hospital mortality, SOFA scores, vasopressor duration ↓	Marik et al., 2017
Critically ill	Standard/standard + Ve + Vc	294/301	1 g every 8 h, 28 days or until ICU discharge	102 ~ 160	Multiple organ failure↓ mechanical ventilation duration↓ ICU stay length↓	Nathens et al., 2002
	Standard/standard + NAC + Vc	32/35	3 g/24 h, >10 days, until ICU discharge or death	Data not shown	lipid peroxidation↑ APACHE II score, sepsis incidence, mortality rate, oxidative stress↓	Sandesc et al., 2018
ARDS	Norepinephrine vancomycin piperacillin/tazobactam + Vc	1	50 mg/kg/6 h, 4X2	Data not shown	Inflammation ↓ alveolar fluid clearance↑	Bharara et al., 2016
	Vancomycin, piperacillin- Tazobactam levofloxacin + Vc	1	0.2 g/kg/24 h, 7; 0.1 g/kg/24 h 8th day; 0.05 g/kg/24 h 9th day	Data not shown	completely recovered	Fowler III et al., 2017
Virus HCV EBV CHIKV Herpes zoster	Antioxidative 7 oral + 4 IV preparations	50	2 g oral tid 20 wks 10 g IV twice weekly	Data not shown	Histologic improvement ↑ ALT↑ viral load↓	Melhem et al., 2005
	Vc	35	7.5–50 g 1 or twice weekly, 24 ~ 243	5000 ~ 8800	EBV EA IgG, EBV VCA IgM↓	Mikirova and Hunninghake, 2014
	Vc	1	100 g/day, 2	Data not shown	C-reactive protein ↓ All symptoms resolved	Gonzalez et al., 2014
	basic analgesic and viral-static therapy + Vc	2	15 g every other day, 12	Data not shown	Neuropathic pain total remission, cutaneous lesions remission	Schencking et al., 2010
	basic analgesic and viral-static therapy + Vc	67	7.5 g 2–4 times/wk, 14	Data not shown	pain scores, hemorrhagic lesions, and the number of efflorescences↓	Schencking et al., 2012
	Standard/standard + Vc	42/45	5 g every other day, 6	Data not shown	No change acute pain postherpetic neuralgia↓	Kim et al., 2016
Myomectomy	myomectomy /myomectomy + Vc	50/52	2 g during surgery, 1 g post operation	Data not shown	blood loss, operation time, hospitalization days ↓	Pourmatroud et al., 2012
	Saline/saline + Vc	25/25	2 g during surgery	Data not shown	No change blood loss, operation time	Lee et al., 2016
Burn	RL/RL + Vc	18/19	66 mg/kg/h, 1	<540	Resuscitation fluid volume, body weight gain, wound edema↓	Tanaka et al., 2000
	RL/RL + Vc	16/17	66 mg/kg/h, 1	Data not shown	fluid requirements↓ urine output↑	Kahn et al., 2011
Allergy	non-allergy + Vc/allergy +Vc	70/19	7.5 g/h, 1h	Data not shown	Serum histamine concentration↓	Hagel et al., 2013

*NAC, n-acetylcysteine; Lo–Vc, low vitamin C group; Hi-Vc, high vitamin C group; SOFA, Sequential Organ Failure Assessment; APACHE II, Acute Physiology and Chronic Health Evaluation II; ARDS, acute respiratory distress syndrome; HCV, hepatitis C virus; ALT, Alanine aminotransferase; EBV, Epstein–Barr virus; EBV EA, EBV Early Antigen; EBV VCA, EBV viral capsid antigen; CHIKV, Chikungunya virus; RL, Ringer lactated solution; Vc, Vitamin C; Ve, Vitamin E; ↑ increase, ↓ decrease*.

## Future prospects

### Low vitamin C levels in *H. pylori* infection: potential mechanisms

Ascorbate concentrations are lower in *H. pylori* infection, probably because of insufficient vitamin C ingestion and corresponding down-regulation of vitamin C concentrations tight control mechanisms including less bioavailability, impaired stomach transport or secretion, and *H. pylori*-associated oxidants accelerating ascorbic acid, or DHA degradation (Woodward et al., 2001; Annibale et al., 2003; Henry et al., 2005; Levine et al., 2011; Aditi and Graham, 2012). Woodward et al. compared a large number of subjects with/without *H. pylori* infection and suggested that systemic bioavailability of ascorbic acid in patients with *H. pylori* was impaired and not related to diet (Woodward et al., 2001). Henry et al. found that proton pump inhibitor omeprazole (40 mg/day, 28 days) decreased plasma vitamin C level in both H. pylori positive and negative subjects with similar ascorbate dietary intake, and indicating a reduced bioavailability of dietary vitamin C (Henry et al., 2005). Alternatively, the lower plasma vitamin C concentrations in patients with *H. pylori*-infected gastritis after eradication may be the consequence of increased active transport of ascorbic acid to regain the high ratio of gastric juice to plasma ascorbic acid (Annibale et al., 2003). In addition, *H. pylori* infection is an inflammatory process producing great amount of ROS; therefore, it is also possible that ascorbate utilization increases in inflammation (Ellulu et al., 2015). Insufficient vitamin C ingestion might be easily avoided through more vitamin C supplementing with pills or from vegetables or fruits (Woodward et al., 2001; Henry et al., 2005). The decrease in plasma vitamin C induced by *H. pylori* infection and/or omeprazole depends less intestinal absorption or more renal leak (Woodward et al., 2001; Henry et al., 2005). To characterize transport or secretion of ascorbic acid from plasma into gastric juice directly (not just assuming active secretion of ascorbic acid from high gastric juice:plasma ascorbic acid ratio), It is worthwhile to further investigate how *H. pylori* infection (inflammatory molecules or *H pylori*'s virulence factors) or medicine affect the function of vitamin C and DHA transporters (Figure 1).

### Pharmacologic ascorbate on *H. pylori* eradication: *H. pylori* antibiotic resistance

As mentioned above, combined oral vitamin C as high as 5 g with standard antibiotic treatment on *H. pylori* infection, the results of *H. pylori* eradication were controversial; the saturated plasma ascorbic acid concentration with oral intake around 100 ~ 300 μmol/L may not be high enough for *H. pylori* eradication (Table 3; Levine et al., 1999, 2001). To be applied to cancer treatments or other diseases, pharmacologic ascorbate as a treatment were easily and safely reached up to 25 ~ 30 mmol/L in blood; and the concentration of H_2_O_2_ at ~ 200 μmol/L as a pro-oxidant drug induced by pharmacologic ascorbate, which was 100-fold of those concentrations that regulate normal cellular processes (Tables 4, 5; Padayatty et al., 2004; Stone and Yang, 2006; Levine et al., 2011; Parrow et al., 2013). Antibiotic treatments are still the primary methods to eradicate *H. pylori*; however, this strategy has been hampered by the recent emergence and spread of *H. pylori* antibiotic resistance in most countries worldwide with frequent treatment failures in at least 10–20% of patients (Pal et al., 2011; Megraud et al., 2013; Camargo et al., 2014; Thung et al., 2016). Taken together, pharmacologic ascorbate may be an obvious addition to existing antibiotic therapies for synergy treatment on *H. pylori* infection. The hundreds-fold elevated concentration of plasma vitamin C and H_2_O_2_ may be especially useful for eradication of *H. pylori* with multiple antibiotic resistances. If it worked as in cancer treatment, pharmacologic ascorbate would play synergic role to cope with *H. pylori* antibiotic resistance and reverse the low ascorbic acid concentrations in blood and gastric acid induced by *H. pylori* infection.

## Conclusions

Current clinical data of *H. pylori* infected gastritis suggested a typical example of vitamin C concentration-function relationship among less vitamin C intake, low ascorbic acid concentrations in gastric juice and plasma, and relevant pathological outcomes of gastric diseases. *H. pylori* eradication had an inverse association with vitamin C concentrations in gastric juice and plasma. In contrast, oral ascorbic acid supplement with or without standard antibiotic treatment on *H. pylori* eradication yielded controversial data. The route of vitamin C administration, orally or intravenously, is critical for plasma ascorbate concentration with two orders of magnitude difference. Intravenous vitamin C, also termed pharmacological ascorbate could achieve 25 ~ 30 mmol/L and form high concentration of H_2_O_2_ as a pro-oxidant drug, which was been extensively used to treat and prevent many disorders like various cancers and other diseases. With all these knowledge and research progress, it is worthwhile to include pharmacologic ascorbate with or without standard antibiotic treatment on *H. pylori* eradication, especially for *H. pylori* with antibiotic resistances.

## Author contributions

HM and HT did literature research, wrote the manuscript, and read and approved the final manuscript.

### Conflict of interest statement

The authors declare that the research was conducted in the absence of any commercial or financial relationships that could be construed as a potential conflict of interest.
